# Touch and relate: body experience among staff in habilitation services

**DOI:** 10.3402/qhw.v9.21901

**Published:** 2014-02-20

**Authors:** Görel Råsmark, Bengt Richt, Carl Edvard Rudebeck

**Affiliations:** 1 Research Unit, Kalmar County Council, Sweden; 2 Department of Health and Society, Linköping University, Linköping, Sweden; 3 Institutet for Samfunnsmedisin, Tromsø University, Tromsø, Norway

**Keywords:** Body experience, habilitation, bodily empathy, intentionality, intercorporeality

## Abstract

In habilitation centres staff meet children with different impairments, children who need extensive support and training while growing up. A prevailing biomedical view of the body in habilitation services is gradually becoming supplemented by a perspective on the body as constantly involved in experiencing and communicating, the latter involving also the bodies of the therapists. Investigating body experience in habilitation staff in their encounters with the children may provide concepts that make it easier to reflect on what is going on in the interaction. When shared among larger number of peers and supported by further research in the field, reflected body experience may become a substantial aspect of professional self-knowledge. Our aim with this study was to contribute to the understanding of what it means to be a body for other bodies in the specific relational context of child habilitation, and more specifically to investigate what role the therapists’ body experience may play for professional awareness and practice. In the study, five physiotherapists and three special-education teachers spoke of physical and emotional closeness (*the body as affection*) but also of a provoking closeness (*the body as provoked*) with the children and of how their own body experience made them more attentive to the children's experience (*the body as reference*). Situations that included bodily limitations (*the body as restriction*) were described, as were situations where the body came into focus through the gazes of others or one's own (*the body as observed*). The body was described as a flexible tool (*the body as tool*), and hands were given an exclusive position as a body part that was constantly communicating. Three shifts of intentionality that form a comprehensive structure for this body experience were discerned. When professional reflection is evoked it may further body awareness, deepen reflection in practice and strengthen intercorporeality.

Staff in Swedish habilitation centres meet children who need extensive support and training while growing up. Since habilitation is integrated into the health care system its practice is dominated by the biomedical tradition (Carlhed, 2007). In clinical meetings attention is often drawn to body parts, their constituents and functions although the child is first and foremost there as a whole person, with a body that is very clearly an aspect (Engelsrud, 1990; Mulderij, 1996, 2000) and so is the therapist. There are two body realities in the clinical encounter: the intersubjective—the intercorporeal—and lived, and the abstract of observation and adjusting interventions. The study started from the assumption that with a better understanding of the conditions of bodily intersubjectivity between therapist and child, the interaction between them might be improved through deliberate measures. To ground this assumption, we need first to visit some of the philosophical discussions about different aspects of body experience.

## Body experience, body consciousness and body awareness

As said by many, but very clearly demonstrated by Drew Leder in his essay “The tale of two bodies” (1992), in biomedicine the model of the body is machine-like: matter set to work by energetic processes. The everyday experience that one's own body is evidently involved in the experience of living, as an aspect of it, is mainly neglected in scientific medicine, nor has it been dealt with seriously in philosophy until the emergence of phenomenology. Here the French phenomenologist Maurice Merleau-Ponty (1947/1964) has become the front figure. According to him, being and consciousness are grounded in the lived body (corps propre) through pre-reflective perception. The lived body is the primordial subject. Body experience per se, however, is not a big topic of Merleau-Ponty's. From his perspective, experiencing the body itself tends to become equivalent to objectification: observation rather than living. The lived experience of the body, which precedes objectification, is “surpassed.” The lived experience of the body was in fact addressed in earlier phenomenology, but without becoming a major field of investigation. Edmund Husserl (1936/1970) leaned on the distinction made in German between “Körper”—the material body, and the living body—“Leib”, that is given to the subject in perception.

In our time Richard Shusterman (2008), a pragmatic philosopher, discusses and describes how developing a basic body consciousness into reflective awareness of the body—somaesthetics—through practice may contribute to self-knowledge and self-cultivation. Without a primary experience of the living body as such, developing somaesthetics would not be possible. From his pragmatic angle regards Merleau-Ponty's body philosophy as an ontology of the human being rather than a phenomenology of body experience and body consciousness.

Responding to Shusterman, J Scott Jordan (2010) suggests that the fact that Merleau-Ponty did not himself investigate the lived experience of the body does not mean that he would have objected to the possibility of higher levels of soma awareness. It is quite evident that Merleau-Ponty has inspired much research in medicine and caring sciences about the lived experience of symptoms and disease. Charmaz (1991, 1995), Corbin and Strauss (1967), Toombs (1993) and Bury (1982) have all made pioneering work on the process of adaption to chronic illness.

Other researchers have studied the bodily and existential implications of distinct diseases or symptoms (Afrell, Biguet, & Rudebeck, 2007; Bullington, 2009; Lundgren & Bolund, 2007; Reventlow et al., 2006). From the perspective of the clinical world, Rudebeck (2001) describes “existential anatomy” as the logic of the lived experience of the body with the intentional horizons: outwards meeting the world, inwards the physical body, and reflexive, where the body as self emerges. The latter would then be the intentionality of somaesthetics and include distinct perceptions such as those of emotions or breathing, or of distinct symptoms, as well as the attention to whole body habits such as walking and balancing, and including the impairment of these faculties. Important to underline is that in the perspective of lived experience, even distinct experiences are aspects of the whole.

In part, Shusterman builds his argument on his own expertise from physiotherapeutic traditions such as Feldenkreis and Alexander therapy. He coined the term somaesthetics to underline the connotations to “a living, feeling, and sentient body”, but “body awareness” is probably the most widely applied term in approaches of physiotherapy that aim at increased body awareness as a path to improved health (Mehling et al., 2011).

### Bodily empathy and intercorporeality

The definitions of body awareness are, however, not exact, and the methods of its assessment vary (Roxendal, 1985). When one embarks on the clinical interaction in diagnosis and therapy, the role of body- or soma awareness for the empathic sharing of body experience comes within focus (Rudebeck, 1992, 2001). Bodily empathy in the clinic is about the understanding of distinct experiences of the “dysappearance” of the physical body (Leder, 1990) and about the experience of disruption or impairment of body experiences and faculties. Bodily intersubjectivity is still not restricted to the field of bodily meaning but plays a major role in social interaction as the interplay of body action, or “body techniques” between people (Crossley, 1995; Mauss, 1979). Here, the concept referred to is usually “intercorporeality”, also introduced by Merleau-Ponty (1947/1964, 1964/1968) but adopted and theoretically developed mainly within sociology, anthropology and feministic philosophy (Crossley, 2006; Csordas, 2008; Goffman, 1959, 1963, 1967; Weiss, 1999). In intercorporeality the intentionality is not one's own or the other's body experience, but the other person as met in a certain context, and the body partakes fully in the intentional expression. One is “a body for other bodies” (Frank, 1995). But for therapists in child habilitation for instance, to imagine the consequences of restrictions of a child's body expression, the mode has to be sensing and listening rather than expressive.

### The therapist–child relationship

Impairments are restrictions in the child's relation to its world. In their long-standing interaction the therapist and child develop a bodily companionship which very probably influences the child's long-term body experience.

Mulderij (1996) has studied body experience and the lifeworld in children and young people with physical impairments. He describes a body that children perceive as rebellious, unreliable, sensitive, dependent, conspicuous, but also vital. In a category called “the body in therapy” the body is sketched as needing constant maintenance. It thus becomes a central theme in these children's lives.

Meeting children with extensive motor impairments is not part of most people's everyday experience. It implies challenges to empathy, not least in its bodily sense. Bodily empathy (Rudebeck, 2001) depends on common references, but the very idea of intersubjectivity presumes also that individuals differ and, therefore, that it always takes imagination to go beyond one's own experience to approach another person's (Stein, 1917/1989; Thompson, 2001). Empathy is always a possibility, but it may be more or less difficult to attain, and the richness of what is shared varies. In habilitation, the therapist needs to see the child's everyday activities and relations from a position close beside him or her, rather than just observing and making inferences from the medical knowledge about the child. With such a stance, functional diagnosis, training and relation become a unity rather than separate projects. Being together is also, in the concrete, communicating about the faculties and limitations of the child. The reference for bodily empathy offered to the therapist is her own, and in terms of faculties usually average, body. The average body is a taken-for-granted executor of bodily intentions. Body experience is of course the ultimate precondition for empathy, but one's own experience of a non-problematic body may become an obstacle if the difference from the child partially blocks attention. Conversely, an ability to surpass one's own privileged situation to expand the field of bodily intersubjectivity would indicate developed professional skills and may aid the therapist in becoming more sensitive to the child's outlook and needs in treatment situations. The therapist then has the means of adapting actions more deliberately to the unique individual and to support the child's involvement and cooperation in treatment and training. How habilitation staff experience their own body in the interaction with the child is therefore a matter of professional importance. It is acknowledged that therapists’ body awareness is important for the effectiveness of therapy (Helvik Skjaerven, Kristoffersen, & Gard, 2010), an understanding that calls for further studies. Ryding et al. (2000, 2004) have found an apparent variability in body awareness as measured and described in interviews among general practitioners but, to our knowledge, except for one very original paper (Santana & Jorge, 2007), there are no empirical investigations among health care staff of the role of one's own body awareness for bodily empathy or intercorporeality.

The present study focuses on how habilitation staff experience their own bodies in the clinical situation. Investigating body experience in habilitation staff in their encounters with the children may provide concepts that make it easier to reflect on what is going on in the interaction. When shared among a larger number of peers, and supported by further research in the field, reflected body experience may become a substantial aspect of professional self-knowledge. Our aim with this study was to contribute to the understanding of what it means to be a body for other bodies (Frank, 1995) in the specific relational context of child habilitation, and more specifically to investigate what role the therapists’ body experience may play in professional awareness and practice.

## Method

The article is based on individual and group interviews with staff at three habilitation centres. Habilitation centres offer families multi-professional teams seeking to meet habilitation needs on an individualized basis. Systematic Text Condensation (Malterud, 2012) was used in the subsequent analysis.

### Participants

Staff at three local habilitation centres in the southeast of Sweden were interviewed. Two of the centres are in sparsely-populated areas, and one is in a major city. In densely-populated areas health care is specialized and habilitation staff mostly meet children with motor impairments and differing syndromes. In the countryside, staff may also meet children with chronic diseases and neuropsychiatric disorders.

The participants, five physiotherapists and three special-education teachers, were all female. The group with the physically closest and longest-lasting contact with these children was the physiotherapists, representing the medical field. Physiotherapists often encourage a focus on the body, seeking to improve motor proficiency, whereas special-education teachers, representing educational theory and practice, point more to child involvement in different activities. Theoretically, these vocational groups represent two important perspectives on the lived body and were chosen in order to ensure variation. There is a body-close, functional perspective and an interactive perspective where the child's communication is supported. In close teamwork, these two perspectives are supposed to be shared in daily practice. Our aim when interviewing both groups was to include a possible range of variation in experience rather than to discern differences. The participants represented vocational careers of between 1 year and over 30 years in the habilitation services.

### Ethical considerations

A written description of the study was sent to three managers of local habilitation centres. With their consent, physiotherapists and special-education teachers in these centres were introduced to the study through a cover letter describing the project, voluntariness regarding participation and the right to end participation at any time. Participants were assured of confidentiality.

Ethical implications were considered, but nothing was discovered that could possibly affect the participants’ reports or their well-being.

The study was approved by the Regional Review Board in Linköping, Sweden.

### Data collection

Information was collected with open-ended interviews and in three steps, with intermediate periods of analysis and consideration of how to proceed. In the cover letter participants were first informed about the aim of the study, to probe body experience. At the start of all interviews the aim was again introduced and participants were briefly guided to think about the subject and to take their time. The interview guide—with questions probing body experience—was used mainly as a list of suggestions and was refined and developed through the first two sampling steps. The same questions were used for both vocational groups, accounting for both professional and individual contributions. Participants commented on their lack of experience in wording thoughts on body experience, consequently putting extra demands on the interviewer to adjust to individual communication abilities. Individual interviews were carried out in steps I and III. Between these a group interview, step II, was conducted.

In step I, one special-education teacher and one physiotherapist both assumed, from the first author's personal knowledge, to give rich reports, were interviewed. Step II consisted of a group interview with six participants. These were then interviewed individually within 2 months in step III. In the interval, the participants had the opportunity to continue reflecting on matters that had been touched upon.

The group interview was expected to afford common experience, and to start a reflective process which could then be harvested in the individual, more personal, interviews. It also gave an opportunity to further develop the interview guide. Participants in steps II–III were recruited through convenience sampling. The director of a habilitation centre in the vicinity was contacted and asked to distribute an introductory letter about the study to all physiotherapists and special-education teachers. All but one agreed to participate in the group interview as well as in subsequent individual interviews.

Individual interviews opened with a request for a story about a recent meeting with a child. If this story included openings into the study area—the participants’ own body experience and subsequently the interaction between participant and child in a habilitation setting—the interview continued. If the story lacked such elements, the interviewer suggested a situation that could serve as a starting point. Rich information about participant experience within habilitation was also facilitated through questions such as: “When you think of your own body, what is the common thought?” and “In what situations in work do you become markedly aware of your own body?” The participants were encouraged to talk openly about their experience, to open up regarding their concerns and to expand their accounts. Probing questions such as “Would you give me an example …?” were used and the participants were encouraged to feel at ease with prolonged pauses in order to give time to find the right words for their experience, and for reflection. The interviewer sought to create the atmosphere of “a reflective space” where the participants could feel free to express themselves.

The group interview started with general reflections on body ideals, followed by a focus on body experience pertaining to work situations. The main question in the interview guide was: “When—and how—will your own body come forward in everyday work?” During the interview, it became obvious that—although the pre-formulated questions were helpful—it was not through them that participants gave the richest reports: it was in their narratives, stories involving meeting with children, that associations were aroused and shared.

The interviewer repeatedly had to return to the main issue of body experience since the participants, drawing on each other's experience, highlighted professional demands and their concerns about “doing a good job.” In the prevailing colloquial atmosphere, participants gave the interviewer new ideas on how to ask about body experience. For the concluding individual interviews, in step III, there was no reason for further changes in the guide.

All interviews were conducted by the first author to whom the participants were little or not known. A few participants had been encountered briefly in professional settings. Interviews lasted 60–90 minutes and were digitally recorded, except for one of the interviews in step I where field notes were taken. The notes included passages of almost exact wording. They have been used in analysis but were not amenable to quotation. The first author did verbatim transcriptions.

### Analysis

Phenomenological philosophy and research on the human body guided the researchers throughout the study, providing “a direction in which to look”, but not determining what to see. This means that our focus was on body experience as an aspect of self-experience rather than on detached body sensations, and on the accounts of experiencing in distinct situations rather than on general reasoning. In our pre-understanding, we also conceived of body experience as varying according to situation and context (Rudebeck, 2001). Therefore, we did not aim primarily to achieve a uniform eidetic structure—the essence—within which variation may occur (Husserl, 1936/1970; Malterud, 2012) but rather, to describe the examples of variation within the context of child habilitation in their concreteness. We found Systematic Text condensation (Malterud, 2012) suited for this purpose while providing a tool for dealing with transcripts in both an open and systematic way. This method takes its departure in Giorgi's psychological phenomenological analysis (Giorgi, 2009) but deliberately refrains from making out the essence of a certain phenomenon in favour of depicting its variation.

The general phenomenological attitude (Malterud, 2012) and the use of “bridling” (Dahlberg, Dahlberg, & Nyström, 2008) served as constant reminders of the importance of trying not to impose a pre-formulated theory onto participants’ accounts. All the way from the initial interviewing to the final stages of analysis, openness and the ambition to stay close to the experience of particular phenomena, was pursued.

The main and the third author initially read through the transcriptions of the eight individual interviews as well as the group interview. This procedure aimed at getting an overall impression and resulted in tentative themes. Material not pertaining to the themes was excluded, whereas sequences of text bound together by a common meaning were noted as units of meaning and later coded. Codes capture the common meaning on a first level of transformation and in this case they are compared and elaborated in order to reach a higher degree of clarity. The codes were subsequently joined in categories that were treated likewise with repeated adjustments. Tentative categories needed to be continually compared, contrasted and renamed to gel into pertinent descriptions. The tentative categories as well as notes written in the coding process were continuously discussed between the first and third author and the second author entered the process to contribute to the final shaping of categories. Research seminars and a group of clinicians, including special-education teachers and physiotherapists, contributed with suggestions for further refinement of the categories throughout the analysis.

The categories that describe participant experience are illustrated by short quotations with fictitious names.

## Findings

The analysis resulted in six categories of *body experience*, namely experience of the body as affection, as provoked, as reference, as restriction, as observed and as being a tool. This is the content structure, e.g., variations of body experience that were distinguished through the analysis. In the subsequent section Shifts of Intentionality, the dynamics of body experience, is described.

### Categories of body experience

#### The body as affection

For the therapists, physical closeness with the children appeared to be self-evidently included in their daily work. There was, however, an intertwining of physical and emotional closeness, indicating a shared world. When therapist and child came close to each other, the therapist could relinquish the strictly professional attitude and instead let the child in. Participants referred to professionalism as the ability to use occupational competencies correctly, but also to use physical and emotional contact to deepen the relation with children. Physical proximity opened for personal affection. Being stroked on the head by a child or being out of breath and sitting close together on a bench were described as good experiences. Gail, who often referred to the experience of children whom she had met, gradually turned to herself and told about how a young child once reached out for her hand, examined it carefully and did not want to let it go. In this prolonged holding of hands, Gail felt affection for the child, as if she had been the mother:

… the child kept on holding my hand. Wouldn't let go and started to sort of examine it and keep itself busy with it. And I accepted that … I felt as close as a mother or someone else who is close to the child.

To give someone your hand is not just a physical action. It is a welcome into a shared world that therapists, but also children, initiate. In physically close and confidential moments, children sometimes start talking about themselves. It is usually the therapist who takes the initiative for physical contact, but it may also be the child. Bonnie reflected on this: “When they [the children] touch [me] that means that they feel secure and they will give something back to me.”

#### The body as provoked

The therapist's body tends to spontaneously withhold itself in situations where children are experienced as intrusive and unaware of body territories. It is a difficult task for the therapist to use her or his own body to establish distance and in the same act to confirm a child. This physical distancing is, however, necessary when the aim is to support the child's own physical integrity. Fanny reflected on her own interaction when showing children how to greet: “It is important, I think, to treat the child in a correct manner. And with a child who also has a mental disability, it is so important to show what a hug should be like.”

Fanny referred to a situation where she very deliberately refrained from a huge hug and instead chose to greet with her hand.

#### The body as reference

What the therapist experiences and has experienced in her own body facilitates understanding of the children with whom she works; of their being in their bodies. The therapist's body may, for example, know what it feels like to be unable to do something one has really longed for or what it means to be in pain. This understanding originates in body experience but may be remembered as a cognitive act. Gail referred to her own experience of how pain can come to rule body and mind:

The fact that it [the body] hurts. And that one does not feel good when it hurts, but gets angry and it is not that nice. And it's just the same with our children when they're in pain, that they can't really be the way they really are. That gives an understanding.

Karen accounted another aspect of bodily understanding when she talked about her body as it was at present as suitable, but also described previous concerns: “One can think about how it was when one was young and there was so much wrong with one's body. And now one can look at a photo and see that there was nothing about that body of mine…so you can understand the children when they think that their bodies aren't good enough… then you have your own experience of disliking your body. Or thinking it's not good enough.” Both therapists referred to an understanding that developed thanks to bodily memories and originating in body experience. There was also an in-the-situation understanding where body experience immediately gave rise to empathetic understanding.

#### The body as restriction

It may happen any time that the therapist's bodily limitations come to the fore. Stiffness and pain may be companions that prompt a move of focus from the child to one's own body. The body is holding back, hindering endeavours and actions. It is difficult to preserve trust in the body when it repeatedly fails to meet ones needs, and when there is a lurking threat of bodily failure. Anne explained how, standing by the pool at the end of a swimming session, she realized that her strength and range would not be enough to lift the limp and wet child.

I think I was very scared that I would not manage to carry him. … I could not get hold of him at all. And I felt that “Oh no, I cannot help him out of this”: fear of not being able to handle things and that he would fall down on the floor.

Fanny recalled a situation where she had just sat down on a low bench, nursing her aching knees and pondering on whether she ought to mention them to the child's parents. Her inner dialogue also held thoughts about the fact that she was just then interacting with a child who would never be able to raise itself from the floor. Fanny felt her body was inadequate. Such an experience may generate a feeling of being at the mercy of the body and its whims and fancies. “These restrictions [of the body] make me humble … for me it's been natural to be able to do this or that. And it's not anymore. Somehow, this is natural ageing.” Ageing was considered a natural process and restrictions were hence accepted.

#### The body as observed

The category “the body as observed” differs from other categories in that it is not as closely linked with the relation to the child. In many situations, therapists act as bodily examples. Parents, assistants or others look at the ways therapists interact with children. Sometimes video recordings are used and the therapist herself will be among those who turn their gaze to their bodies as exposed as objects on the screen. This experience was not described as that of being conspicuous, but rather of being subjected to others’ scrutiny–and one's own. Laura reflected on how difficult it had been to get used to the image of herself on the screen and described the fact:

… that others would sit and watch this [video recording], not only me. So it took quite a while before I could let go. Perhaps it has to do with my body, what I said and what I did, how I moved and what I actually look like.

Other situations also involved the experience of being watched. Bodily exposure could occur at the pool when helping a child to swim. The therapist's body suddenly appeared as a separate entity in consciousness, was observed, valued and sometimes felt as embarrassing. Maureen, when recalling a meeting with the parents of a dying child, told about her own reaction when she found herself crying uncontrollably: “When your body reacts in a way that you cannot cope with, it is then, I suppose, that you feel exposed.” In this attentiveness, where the body is watched by oneself or by others, staff may enter a process of objectifying themselves.

#### The body as tool

The therapist's body was consciously and constantly used to replace toys, training equipment or technical aids. The body as a whole was used although the hands were given a primary position as tools. The role of contact with hands varied. Participants told of a spontaneous use of the hand as well as of a conscious use. Spontaneous hands knew by themselves what to do and followed their own course. In demanding situations, such as when a child had to be held in a different manner, the work of the therapist's hands would attract notice. Such attention, or awareness, could also arise when the hands intended to convey a message. Hands were then not only conscious, but consciously used.

To use one's own body instead of different equipment means that nothing comes between the body and the child. The body becomes a tool, a relating instrument, on behalf of the child. When the body is trusted as such a tool, improvisation and playfulness are readily there. Therapists expressed trust in their own bodily flexibility: they trusted their ability to solve problems as they arose and to meet new challenges. In descriptions of the replacing of physical objects, and the favouring of one's own body as a tool, this process seems quite self-evident; nothing can be as good as using the body in play or when overcoming obstacles. This message was often passed on to parents and assistants.

Karen related that she often had to observe children, but emphasized that using her own body is what she really appreciates when with them:

…I use my body a lot in work; especially with small children where I prefer this to using cushions and such things. My body is a tool here and I try to convey to the parents that their bodies are also tools.

The body is considered as a resource that one has to be careful with. Taking good care of one's body implies that it will also be fit in the future.

#### Shifts of intentionality

When participants told about their experience of encounters with children continuous shifts of the intentionality of body experience ran as an implicit theme through their narratives. The participants’ experience included attentiveness to their own body (self-attentiveness), a self-evident being-in-the-world where the body was taken for granted (immediate relation), as well as situations of conscious reflection on interaction (reflection in relation). Examples in the categories illustrate all three modes of intentionality.

In *self-attentiveness*, the therapist's body comes into focus when her bodily resources fail to meet her needs and also where her body is objectified in the gazes of others or in her own observation. Consciousness turns towards bodily being as it is observed. Therapists get occupied within themselves—the child is gone. Although shifts of intentionality were described mostly as continuous, self-attentiveness was sometimes experienced as a sudden interruption. Participants told of self-attentiveness in situations when the body was experienced as restricted or as observed, but also when the body was experienced as provoked.

In *immediate relation*, the body is immersed in activities where therapists and children are mutually involved and stay in the background. It is not an object of conscious thought, but it is still there since the body is the necessary prerequisite for following one's intentions. A body that is complaisant and flexible facilitates action where attention to it is not in focus. In the present study, the body was involved in an immediate relation when the therapists experienced “the body as affection” and when hands were used in a spontaneous way.

In *reflection in relation* therapists reflect upon their own bodies, upon the body of the child and upon bodily aspects of the relation in a conscious and decided manner. Therapists may seek to leave a message of acceptance through a conscious use of hands. Touching can be filled with acceptance, trust or just the plain message: “I am here with you.” This will happen, for example, when the body is experienced as a tool and when the hand is used in a conscious way. “The body as reference” is also a category where reflection in relation takes place. An example was when participants told about how body experience, such as lacking strength or agility, made them think of specific children, and this furthered both understanding and compassion, qualities that they brought to subsequent meetings. This describes a shift from self-attentiveness to reflection in relation.

## Discussion

### Methodological considerations

In our analysis we relied on verbal presentations that may include lapses of memory and conscious or unconscious changes in the retelling. In addition, giving subjectively accurate words to body experience is not easy and different aspects of body experience were recurrently intertwined in the participants’ reports. This is particularly evident in the categories “body as affection” and in “body as tool.” We believe that the combination of high topical relevance among the participants, the absence of prestige-mindedness during the interviews and the technique of asking participants to talk about actual episodes in their work resulted in experience-near accounts.

The main author, herself a physiotherapist in habilitation services, made deliberate use of her own experience when probing participants’ body experience as well as, later on, in the analysis of the transcripts (Thorne, 2008). The fundamental idea behind the research was that bodies communicate in all human encounters and that bodily interaction is of importance in creating relations. Investigating and describing the interaction between therapist and child necessarily came prior to the clinically important question “how intercorporeality may contribute to successful therapy?”. We regard such an open and descriptive stance favourable in terms of including all kinds of experiences into the analysis and results, also those that were problematic, such as when body closeness became provoking or when the therapist's own body became disturbing or hindering.

As our approach was descriptive, the challenge of bridling (Dahlberg et al., 2008) was mainly about preventing that own professional experience and ideology (first author), besides giving the sensitivity necessary for a deeper understanding, lead to the material being read with prejudiced eyes (Thorne, 2008). Therefore, the two other authors, who are not involved in child habilitation, challenged the first author's coding and categorizations from a general lifeworld position. The ideas and suggestions from the research seminars were assimilated by the first author into her analysis of the material, and in terms of pre-understanding the input from the seminars were never made a separate issue.

The next level of understanding was the phenomenological outlook, with the emphasis on the phenomenology of the body, as described in the method section. As such, the phenomenology of the body is the result of a “bracketing” scrutiny of body experience, but once laid out it also takes the shape of theory, which may preclude a non-prejudiced observation. Here, we believe that the fresh field and the fact that we did not go for the essence of therapist body experience, helped us become informed by our data rather than making it, more or less, the reflection of our pre-understanding. We think that it a first on the eidetic level that influences from phenomenology may act in the direction of conformity of the results.

Performing data collection in several steps as well as data analysis with discussion among authors and the sharing in research groups and in a group of clinicians served to slow down the process. The understanding of body experience among staff in habilitation centres was gradually emerging. This slowness that allowed for ambiguity and contradiction, which was dealt with through discussions among the authors, and reconsiderations among the authors, was also necessary to “bridle” the process of analysis to release the creative potential of the pre-understanding. There was an explicit ambition to stay open before emergent themes and categories.

The possibility of getting disparate data when using both individual and group interviews was considered when planning the study, but in the subsequent analysis no such split appeared. In a group interview, there is a risk that individuals adhere to a “group voice” that makes itself heard at the expense of personal experience or get dominated by compelling group members (Smithson, 2000). No such complications were identified during interviews and analysis, and directly after the group interview, participants spontaneously certified that they felt secure and very much at ease in the group.

Physiotherapists and special-education teachers often referred to different types of factual situation, but still with similar experience and we did not find any indications of unequal contributions to the categories between the two groups. Apart from aspects of ageing body experience was strikingly alike regardless of the length of vocational career reported.

Child habilitation is a gendered activity with few male therapists. In the sample all participants were, as previously mentioned, female. The need for including men was discussed during the procedure, as a possible means to enhance transferability. The inclusion of single male participants would probably not solve the issue and the addition of several would, on the other hand, belie the situation in child habilitation.

Participants were few. The very restriction in the number of protocols offered an opportunity to analyse the material in depth, and the wealth of detail was thus preserved through the process of condensation. We therefore hold the results to be internally valid, and by making the study within a bodily phenomenological and sociological frame of reference, we think that the results are theoretically generalizable to be valid for health care staff also in settings other than child habilitation. The actual drawback of a small study group is the limitation of the scope of experiences within the field studied. Perhaps, with a larger study group it would have would have been possible to identify yet more body experiences characteristic of habilitation staff in interaction with the children. There is a balance between depth and coverage and here we have laid the emphasis on depth.

### Discussion of findings

When recalling specific situations, participants could easily verbalize their body experience and often commented in a vivid and committed way. This readiness to share body experience revealed a deep commitment to the issue, with moral underpinnings, and in agreement with Rudebeck (2001) and Shusterman (2008) it points also to the fact that the attention to body experience is not necessarily about distancing and objectification.

In the following findings are discussed in terms of body awareness, the impetus of touching and relating and the role of shifts of intentionality. The section closes with comments on body awareness on behalf of clinicians and researchers.

#### Increased body awareness

Some of the categories of body experience, such as “the body as restriction” were obvious to the participants all along, while for instance “the body as affection” and the use of hands in “the body as tool” were so closely interwoven with practice that they were surprising when retrieved. In all, intercorporeality, rather than the sensing intersubjectivity, was the dominating frame of reference when recalling the interaction with the children. Here we believe that the interview situation as such, by a mere refocusing of memory, furthered body awareness, in turn making a level of latent body experience available. Besides giving the material for analysis, the interviews thus suggest a method, where the reflection on their interaction with the children may help habilitation therapists improve their body awareness. One objection, or doubt, that may be raised, is that bringing background, or latent, fields of experience into attention may turn into a self-occupation that decreases the spontaneity of the therapeutic interaction. On this point we agree strongly with Shusterman (2008) when he argues that a fuller access to body awareness—somaesthetics—enhances social interaction. Embodied experience and habits also include hindrances e.g. tension, restraint, anxiety and loss of balance that hamper interaction and increase a mostly negative self-consciousness. When one becomes aware of such hindering reactions of one's own body they may be dealt with implying the chance of integrating or overcoming them. In the context of our study a knee problem of the therapist due to arthrosis may either distract her from interaction, or deepen the understanding of the pain a child experiences when a joint is extended to the limit of its motion range, and from there, in all similar situations in the child's living: a restriction turns into an instructive and useful reference for bodily empathy (Rudebeck, 2001). In the study by Gyllensten, Skär, Miller, & Gard (2010) where physiotherapists were included, the participants were asked to describe their experience of body awareness. Body awareness was described as enhancing the ability to interact with others and to participate in society. The body awareness training here referred to was not merely about increased attention through reflection, but included exercises of body awareness. Body awareness is both introspective into experience and expressive (“performative” in Shusterman's vocabulary) through movements and balance (Ryding et al., 2000). We find it probable that body awareness training may add to the bodily interactive and empathic abilities of habilitation therapists. Treatment and training in habilitation include attempts to further awareness in the body (Roxendal, 1985) and playfulness in the body (Sherborne, 2001). In the Relation Play method (Sherborne, 2001), relating to others as bodies represents both means and ends.

#### Touching and relating

Affection and closeness were closely related in the therapist-child relationship. Participants described meeting with children as situations of focused interaction (Goffman, 1963). In these situations, closeness rendered a shared space and cooperation in movement that also was a joy in being together. Participants discovered that touching is also relating. They professed concern about how the mutuality that they appreciated could be preserved, even in e.g. assessment situations where children become bodily exposed. When discerning the relational load of bodily closeness, the sense of responsibility was formulated. An “interaction tonus”, by which Goffman (1963) means an attachment to and regard for the diverse aspects of specific situations, had to be sustained.

This dependence became very clear in the interviews in that closeness was sometimes experienced as provoking and evoked defensive responses, which was a real challenge as the opposite was usually expected. Another challenge to the therapeutic relationship was the sudden and embarrassing self-observation, where the therapist saw herself through the eyes of “the other” (Sartre, 1943/1969). Here a third party was usually involved: the parents, colleagues or doctor. By accepting the fact that the therapist's own person sometimes stands in the way, these situations may be recognized more easily and the readiness to deal with them improved. Sartre regarded “the eye of the other” to be the general way of being aware of the own body, with or without salient emotions involved. Our findings talk differently and are on the line with the views of Shusterman and Rudebeck, referred to in the introduction. For our participants body experience was an important dimension of self-experience. Attaining the eyes of others was a special case, had a clearly social implication and was a position driven by quite strong and negative emotions. Sartre's view was logically inferred more than being based on experience.

Work in habilitation services rests to a great extent on the use of hands. This use can be referred to as a tactile knowing, a knowledge that therapists were only vaguely aware of. An everyday understanding of the concept “tacit knowing” was often referred to (Kontos & Naglie, 2009; Polanyi, 1967). It was appreciated and valued. When hands were doing their job the therapists were focused on the interaction with the child. In some situations body experience was all hand experience, the hand being the only body part that was explicitly mentioned and elaborated. No situations were described in which the hand was apprehended as a technical instrument used in for instance assessments. Such situations were probably so natural and necessary that they were not even mentioned. The body is both living and the pre-reflective tool of its own living (Merleau-Ponty, 1947/1964; Svenaeus, 2000). The passage from the tacit intercorporeality of hands in the therapeutic situation to recalling it in retrospect in “action in reflection” (Schön, 1983/1991) showed that the knowing of many actions was not tacit at all but rather was taken for granted as the fruits of practice and the integration of faculties into the professional repertoire. Increased awareness of these faculties may deepen the bodily relation between therapist and child and admit a conscious choice “to be a body for other bodies” (Frank, 1995). Such awareness may also be very helpful in making the professional skills accessible for deliberate training, and this also includes, of course, technical aspects of the use of the hands.

#### The shifts of intentionality

Immediate relation—the interactive, intercorporeal position was the intentionality mainly referred to when the participants described their body experience when working with the children. The interviews provided an expansion of the reflection in relation in retrospect. This was more a recall of lived experience through body awareness than a construction and objectification. The relation to the child was the background, and the framing, of therapists’ experience of their own body. The implication of this is two-fold. First, the authenticity of the reflection in relation in the interviews suggests that it perhaps plays a more important role in work than spontaneously recognized. It is the necessary platform of responsibility that gives room for judgement, decisions and reconsideration. As such, it is also the prerequisite for the therapist to be able to consolidate the therapeutic relationship when it is challenged by uncomfortable emotions in the intentionality of self-attentiveness. Secondly, reflection in relation, which the interviews invited, may become alerted also to the future. If so, and if adopted in this form among many therapists, it may, besides strengthening the professional role of the individual therapist, yield a common basis for reflection and theory in practice of child habilitation (Schön, 1983/1991).

#### The embodied clinician and researcher

Empirical investigations of body experience in health care staff are scarce and so are investigations or discussions on its role in sharing the experiences of patients. Our findings indicate that this is an important field of investigation. For physicians, “bodily empathy” emphasizes the shared bodily existential conditions as a necessary prerequisite for their grasp of patients’ symptom presentations (Rudebeck, 1992). The study by Ryding et al. (2004) demonstrated substantial differences in body awareness among general practitioners. Training to enhance body awareness/somaesthetics among health professionals may in fact be a project of general interest. In her very personal study, Santana and Jorge (2007) discuss how the sense and meaning of one's own body may help in assisting others in dying. Ellingson (2006) argues that the awareness of one's own body is also a matter for researchers themselves, an opinion that we share as far as the research addresses body experience. The body consciousness developed in our own clinical practice and in body awareness training (first and last authors) and in earlier research is decisive for the pre-understanding of our study.

## Conclusions

This study highlights the importance of child habilitation therapists becoming attentive to their body experience when in interaction with the children. During interviews participants repeatedly reported that they had become aware of body experience that they hitherto had not had access to. They explicitly expressed an urge to share their experience with colleagues and listen to narratives and reflection from others. When professional reflection is evoked it may further body awareness, strengthen intercorporeality and deepen reflection in practice. The results of this study, the categories of body experience within the shifts of intentionality, may provide a useful conceptual framework for professional reflection. Therapists have the opportunity to link body experience in the clinic, as expressed in the categories, with the different shifts of intentionality. Such linking may further a deeper understanding of intercorporeality and make therapists more attentive in situations where, for example, bodily provocation and self-attentiveness coincide and may threaten the relationship between therapist and child. How therapists, through deliberate measures, may improve interaction has to be explored in clinical practice and sharing between colleagues, as well as in future research.

The study does not provide insight into how interaction actually turns out in meetings. Observational studies could highlight bodily interaction and render new dimensions to the concept of intercorporeality. It would be of special interest to observe how staff uses their hands, these physically and emotionally touching instruments of ours.

Future studies are needed to explore how professional reflection on body experience becomes a self-evident part of daily practice and how skills in interaction can develop into a common basis for reflection and also be accessible for deliberate training.
